# Domain-specific effects of physical activity on the demand for physician visits

**DOI:** 10.1007/s00038-020-01376-5

**Published:** 2020-05-06

**Authors:** Simon Spika, Friedrich Breyer

**Affiliations:** Department of Economics, University of Konstanz, Box 135, 78457 Konstanz, Germany

**Keywords:** Physical activity, Leisure time physical activity, Occupational physical activity, Health care utilization, Physician visits, Konstanz Life-Study

## Abstract

**Objectives:**

To assess domain-specific effects of physical activity (PA) in the relationship with health care utilization and to investigate whether a measure that aggregates PA across domains (leisure, transport, work) is appropriate.

**Methods:**

Data were retrieved from a longitudinal cohort study conducted in Southern Germany (women *n* = 1330, men *n* = 766). The number of physician visits was regressed on total PA and on PA differentiated by the domains leisure time, travel time and working time in a negative binomial model.

**Results:**

For women, no association with physician visits is found for total PA, while high leisure time physical activity (LTPA) is associated with 22% more visits. The effect of high LTPA is statistically different from the effect of high total PA. For men, no significant associations are found for both measures.

**Conclusions:**

The specific, positive effect of high LTPA on physician visits among women shows that using an aggregate measure of PA is inappropriate for analyzing the relation between PA and health care utilization. Further, the positive relationship should be considered in attempts to promote physical activity.

**Electronic supplementary material:**

The online version of this article (10.1007/s00038-020-01376-5) contains supplementary material, which is available to authorized users.

## Introduction

Physical activity (PA) has been proven to be an important factor in the prevention of numerous chronical diseases (Bull et al. 2004; Maruti et al. 2008; Meisinger et al. 2007; Sundquist et al. 2005), and the biological mechanisms behind these benefits are well understood (Bouchard et al. 2012; Bull et al. 2004). As with each prevented case of illness the associated use of health care resources is avoided, PA is seen as a key modifiable factor in the struggle against ever rising health care expenditures.

However, besides these effects, which pertain more to a long-term relationship, there are also direct associations, some of which also increase contemporaneous health care utilization. Examples are adverse events of sport activities like injuries, muscular damage, weakening of the immune system due to highly intense training (Lynch et al. 2019) and psychosomatic symptoms like eating disorders (Verhagen et al. 2012). In fact, evidence on this association is less clear. Although a recent review suggests that a negative relationship between PA and health care expenditures is found in the majority of studies (Ding et al. 2017), no significant association (Chevan and Roberts 2014; Karl et al. 2018) or a positive relationship (Kang and Xiang 2017; Lynch et al. 2019) is reported in some cases. Ding et al. (2017) argue that it is problematic to compare the estimates of the effect of PA, partially because of methodological differences between studies. An important issue is how the measure of PA actually is defined. Specifically there is no consensus how the different domains (i.e., leisure time, travel, transport) are considered in the measure of PA. This gives rise to the question whether PA needs to be assessed for each domain separately or whether it is sufficient to assess total PA aggregated across all domains, as it is done for example in the US Medical Expenditure Survey MEPS (MEPS HC AP) analyzed in several studies (Kang and Xiang 2017; Valero-Elizondo et al. 2016). Clearly, if it does not matter for the effect of PA in which domain it is exerted, assessing total PA without differentiating by domain is sufficient. But if for example PA during occupation time does not have the same health effects as PA during leisure time, as argued by Holtermann et al. (2018), a measure which aggregates physical activity across domains is not suitable.

Domain-specific associations with the risk of various health outcomes are addressed in a number of studies, e.g., obesity (Abu-Omar and Rütten 2008; Chu and Moy 2013; Petermann-Rocha et al. 2019; Sarma et al. 2015), high blood pressure (Chu and Moy 2013; Petermann-Rocha et al. 2019; Sarma et al. 2015), elevated blood glucose or diabetes (Chu and Moy 2013; Petermann-Rocha et al. 2019; Sarma et al. 2015) or heart disease (Sarma et al. 2015). Overall evidence from these studies suggests that leisure time PA is associated with a lower risk for these outcomes, while occupational PA is less likely to reduce the occurrence. Likewise in a meta-analysis of over twenty prospective studies, Li et al. (2013) report a negative effect of leisure time PA, but a weakly positive effect of occupational activity on the risk of cardiovascular disease.

In contrast, studies addressing domain-specific effects of PA on effective health care utilization or expenditures are rare. An exception is the study by Codogno et al. (2015), where PA was assessed on the three domains occupation, sport and non-sport in a population of about 1000 patients aged 50 years or over, registered in Brazilian health care units. Codogno et al. (2015) analyzed the association of PA in the different domains with different kinds of health care expenditures and found that occupational activity and sport were negatively associated with drug expenditures, while for overall expenditures a significant negative association is reported with non-sport activities. Clearly not only because of the specific population analyzed by Codogno et al. (2015), more evidence on the domain specific effects of physical activity on contemporaneous health care utilization is needed.

The aim of this study is to assess and to compare the association of PA with contemporaneous health care utilization, namely the number of physician visits for the population of a German cohort study, when PA is considered either with a measure that aggregates across domains or with a measure that differentiates between PA in different domains.

## Methods

### Data sample

The data were retrieved from the Konstanz Life-Study, a longitudinal cohort study for individuals aged 18+ conducted in the region of Konstanz, a mid-sized city in southern Germany. The study was launched in spring 2012 as part of the EATMOTIVE project funded by the German Federal Ministry of Education and Research. For the Konstanz Life-Study data were collected at a central site (city center), participants were recruited via flyers, posters, and newspaper articles (Klusmann et al. 2016). Due to data requirements, for the present analysis the observations from waves 2016 and 2017 are used in a pooled sample, encompassing 2096 observations.

### Dependent variable

The number of visits to any physician (including specialist) in the last 3 months before the date of the interview was used as an indicator for outpatient health care utilization. This definition is similar to the one formulated in the representative German Socio-Economic Panel (SOEP) (Reber et al. 2018; Greiner et al. 2018). The time span of 3 months is frequently used for nonurgent ambulatory visits to minimize recall bias and underreporting (Bhandari and Wagner 2006).

### Explanatory variables

PA was measured with the Global Physical Activity Questionnaire (GPAQ), which was developed by the WHO as an instrument to monitor physical activity at the population level (Bull et al. 2009; WHO 2016). The GPAQ has been shown to have reasonable reliability (Bull et al. 2009; Riviere et al. 2018) and, as compared to the more widely used International Physical Activity Questionnaire (IPAQ) as well as to accelerometer assessed physical activity, reasonable validity (Cleland et al. 2014; Riviere et al. 2018). With the GPAQ, the participant is asked how much time he or she spends in a normal week on moderate and vigorous PA during leisure-time (LTPA), work-time (WTPA) and for the time spent with walking or biking for travel purposes (TTPA). We assigned each observation to one of the three activity levels “highly active,” “moderately active” or “low active” according to the criteria defined by “IPAQ analysis framework” (Sjöström et al. 2006; Wallmann-Sperlich and Froboese 2014). To be “highly active” an individual either must exert vigorous PA on at least 3 days per week accumulating at least 1500 MET-minutes (the equivalent energy expenditure associated with 1500 min sitting) or must exert any combination of walking, moderate or vigorous PA accumulating at least 3000 MET-minutes per week. A “moderately active” individual exerts either vigorous PA of at least 20 min on 3 or more days per week or moderate PA and/or walking of at least 30 min per day on 5 or more days or any combination of walking, moderate or vigorous PA activities on 5 or more days achieving a minimum of at least 600 MET-minutes/week. The category “low active” entails individuals not meeting any of the criteria for the “highly active” or “moderately active” level and also includes individuals exerting no PA at all.

Based on this categorization, we derived two measures of PA. The first measure is suggested by the IPAQ guidelines and categorizes individuals according to total PA aggregated across the three domains. With the second measure, in contrast, each observation is assigned to three domain related categories, according to PA achieved in the single domains. For example, an individual who achieved 600 MET-minutes with three bouts of at least 20 min vigorous activity during leisure time but who exerts no TTPA or WTPA is assigned to the moderate LTPA category and to the low active category for TTPA and WTPA. The categorization according to the IPAQ guidelines is most suitable for the main purpose of this study. Because it relies not only on energy expenditure or time—as suggested, e.g., by the WHO recommendations (WHO 2016)—but also considers the frequency of PA, and because it comprises three activity levels, it offers sufficient homogeneity on the same activity level between the different domains needed for comparison.

With the differentiated measure, however, the moderate and high WTPA categories contain very few observations (s. Table 1) and we therefore clustered the observations in a single “at least moderate” category. As this category contains moderately and highly active individuals in more or less equal shares, the comparability with, e.g., moderate LTPA as well as high LTPA is impaired. Also the high TTPA category contains very few observations. For practical reasons we assigned those observations to the moderate TTPA category. But because of the negligible number of highly active individuals during travel time in relation to the number of moderately individuals, the comparability to the moderate LTPA category is not impaired in this case.

**Table 1:** Sample description

Variable	Total (*n* = 2096)	Women (*n* = 1330)	Men (*n* = 766)
Age	41.07 (17.16)	39.91 (16.38)	43.1 (18.27)
High school degree	1531 (0.73)	985 (0.74)	546 (0.71)
Occupation			
Employed/self-employed	1133 (0.54)	732 (0.55)	401 (0.52)
Student/vocational training	627 (0.3)	417 (0.31)	210 (0.27)
Unemployed	37 (0.02)	21 (0.02)	16 (0.02)
Retired	263 (0.13)	129 (0.1)	134 (0.17)
Homemaker	36 (0.02)	31 (0.02)	5 (0.01)
Income			
< 1000 EUR/month	490 (0.23)	340 (0.26)	150 (0.2)
1000–2000 EUR/month	425 (0.2)	313 (0.24)	112 (0.15)
2000–3000 EUR/month	403 (0.19)	256 (0.19)	147 (0.19)
3000–5000 EUR/month	478 (0.23)	261 (0.2)	217 (0.28)
> 5000 EUR/month	300 (0.14)	160 (0.12)	140 (0.18)
Kids age < 14	0.12 (0.33)	0.12 (0.32)	0.14 (0.35)
Health status			
Poor/very poor	96 (0.05)	66 (0.05)	30 (0.04)
Fair	386 (0.18)	231 (0.17)	155 (0.2)
Good/very good	1614 (0.77)	1033 (0.78)	581 (0.76)
Phys. visits due to chron. disease y/n	359 (0.17)	230 (0.17)	129 (0.17)
Waist-to-hip ratio	84.86 (11.28)	81.36 (10.35)	90.95 (10.18)
Smoker	317 (0.15)	173 (0.13)	144 (0.19)
Alcohol consumption			
< 100 g alc/week	1668 (0.8)	1136 (0.85)	532 (0.69)
100–200 g alc/week	252 (0.12)	125 (0.09)	127 (0.17)
200–350 g alc/week	133 (0.06)	53 (0.04)	80 (0.1)
2 > 350 g alc/week	43 (0.02)	16 (0.01)	27 (0.04)
No. physician visits	1.46 (1.9)	1.62 (1.96)	1.18 (1.76)
LTPA			
Low	960 (0.46)	636 (0.48)	324 (0.42)
Moderate	319 (0.15)	209 (0.16)	110 (0.14)
High	817 (0.39)	485 (0.36)	332 (0.43)
TTPA			
Low	1088 (0.52)	655 (0.49)	433 (0.57)
Moderate	978 (0.47)	656 (0.49)	322 (0.42)
High	30 (0.01)	19 (0.01)	11 (0.01)
WTPA			
Low	1873 (0.89)	1190 (0.89)	683 (0.89)
Moderate	124 (0.06)	81 (0.06)	43 (0.06)
High	99 (0.05)	59 (0.04)	40 (0.05)
Total physical activity			
Low	235 (0.11)	142 (0.11)	93 (0.12)
Moderate	835 (0.4)	566 (0.43)	269 (0.35)
High	1026 (0.49)	622 (0.47)	404 (0.53)

Means or frequencies (standard deviations or percentages). Data from the Konstanz Life-Study, waves 2016 and 2017, Germany

*LTPA* leisure time physical activity, *TTPA* travel time physical activity, *WTPA* working time physical activity

We controlled for a number of further covariates. Self-assessed health status was included by three dummy variables, indicating whether an individual rates own health as “poor/very poor,” “fair” or “good/very good.” We also included a dummy variable indicating whether the individual had a physician visit due to one of sixteen chronical diseases. In addition we controlled for education (using a binary variable indicating whether the individual has a high school degree required to attend a university, i.e., a degree like the German “Abitur”), gender, age, smoking, alcohol consumption, occupation, income, children aged below 14 years in household, waist-to-hip ratio and a time dummy indicating the year 2017.

### Data analysis

To account for gender differences, the analysis was conducted for women and men separately. To assess the effect of PA on physician visits, we estimated negative binomial models with exponential mean function. Because our dependent variable is overdispersed in the data (i.e., the variance of physician visits exceeds the mean), the negative binomial estimator provides a more efficient estimation than the Poisson quasi-maximum likelihood estimator (Cameron and Trivedi 2013). As there are only 33% observations from individuals who participated in both waves 2016 and 2017, we did not employ panel techniques but report cluster robust standard errors.

We analyzed three specifications of the negative binomial model. All specifications include the same set of control variables, but differ in the variables representing PA. In Specification 1 the aggregate measure of PA is included. The differentiated measure is considered with Specification 2. Specification 3 finally uses the differentiated measure of Specification 2, but additionally includes interaction terms between the LTPA and TTPA on the respective levels. With Specification 3 we are able to identify the “pure” domain effects, because the regression coefficient for a particular domain/level category (e.g., moderate LTPA) represents the effect for an individual who is active on this particular domain but only low active on the other two domains. We used the Chi-square Wald test to test whether the coefficient estimates for PA in the single domains in Specification 3 are different from the coefficients for total PA in Specification 1. Since the aggregate measure in Specification 1 assumes that it does not matter for the effect of PA on which domain it is actually exerted, the estimates should not be different in both specifications.

All calculations were carried out with R version 3.6.1.

## Results

### Descriptive analysis

In our sample 1330 (63%) observations are female, 776 (37%) are male. The mean age for women is 39.9 years, for men 43.1 years. The mean number of visits is higher among women than among men (1.62 vs. 1.18). In the domain of leisure time, 48% of women are low active while 36% are highly active. Men appear to exert more LTPA, as 42% are low active but 43% are highly active. For both genders, the moderate LTPA category contains the lowest number of observations (16% resp. 14%). In contrast, women are more physically active for travel purposes, as 49% of women are in the moderate TTPA category and 49% in the low active category, while 42% of men are in the moderate and 57% in the low active category. Only 1% of women and men are highly active in the travel domain. In the working domain, 89% of women and men are low, 6% moderately and 4–5% are highly active. When PA is aggregated across the three domains, only 11% of women and 12% of men are categorized as low active. In contrast, 43% and 35% are in the moderate and 47% and 53% are in the high activity category, respectively. Table 1 provides the description of all variables used.

### Regression analysis

The estimation results for the coefficient estimates of the PA dummies and the interaction terms of the Negbin models are given in Table 2 (women) and Table 3 (men). The estimation results for all variables are given in Tables S1 and S2 in the supplementary material.

**Table 2:** Estimation results negative binomial model, women

Independent variables	Specification 1	Specification 2	Specification 3
Total PA (ref.: Low)			
Moderate	− 0.03 (0.10)	–	–
High	0.04 (0.10)	–	–
LTPA (ref.: Low)			
Moderate	–	0.11 (0.09)	0.11 (0.14)
High	–	0.15* (0.07)	0.20* (0.09)
TTPA (ref.: Low)			
Moderate	–	− 0.05 (0.06)	− 0.02 (0.09)
WTPA (ref.: Low)			
At least moderate	–	0.01 (0.16)	− 0.37 ^+^ (0.21)
Interactions			
LTPA mod × TTPA mod	–	–	− 0.02 (0.19)
LTPA high × TTPA mod	–	–	− 0.15 (0.13)
LTPA mod × WTPA act	–	–	0.09 (0.29)
LTPA high × WTPA act	–	–	0.33 (0.33)
TTPA mod × WTPA act	–	–	0.36 (0.29)
*n*	1330	1330	1330
Akaike Information Criterion	4473.4	4473.9	4474.7

Dependent variable: number of physician visits. Data from the Konstanz Life-Study, waves 2016 and 2017, Germany

Shown are only estimates for variables associated with physical activity

+*p* < 0.1, *p < 0.05, cluster–robust standard errors in parentheses

*LTPA* leisure time physical activity,* TTPA* travel time physical activity,* WTPA* working time physical activity

**Table 3:** Estimation results negative binomial model, men

Independent variables	Specification 1	Specification 2	Specification 3
Total PA (ref.: Low)			
Moderate	0.02 (0.14)	–	–
High	0.04 (0.14)	–	–
LTPA (ref.: Low)			
Moderate	–	− 0.04 (0.15)	− 0.19 (0.21)
High	–	0.03 (0.11)	− 0.02 (0.16)
TTPA (ref.: Low)			
Moderate	–	0.05 (0.10)	− 0.10 (0.15)
WTPA (ref.: Low)			
At least moderate	–	− 0.16 (0.14)	− 0.35 (0.22)
Interactions			
LTPA mod × TTPA mod	–	–	0.23 (0.28)
LTPA high × TTPA mod	–	–	0.14 (0.21)
LTPA mod × WTPA act	–	–	0.48 (0.43)
LTPA high × WTPA act	–	–	− 0.23 (0.31)
TTPA mod × WTPA act	–	–	0.39 (0.27)
*n*	766	766	766
Akaike Information Criterion	2227.2	2230.3	2235.5

Dependent variable: number of physician visits. Data from the Konstanz Life-Study, waves 2016 and 2017, Germany

Shown are only estimates for variables associated with physical activity

+*p* < 0.1, *p < 0.05, cluster–robust standard errors in parentheses

*LTPA* leisure time physical activity,* TTPA* travel time physical activity,* WTPA* working time physical activity

### Women

In Specification 1 with total PA aggregated across domains the coefficient estimates for moderate and high total PA are low in magnitude and not significant for women (Table 2). In Specification 2, considering PA by domain, both coefficient estimates for LTPA are positive and comparably large in magnitude, but only the coefficient for high LTPA is significant. Because of the exponential mean function in the negative binomial model, the coefficient estimate of 0.15 indicates 16% (= exp(0.15) − 1, CI [+ 2%, + 33%]) more visits for women who are highly active during leisure time compared to women, who are low active in the leisure time domain. Moderate TTPA is associated with 5% fewer visits, which is not significantly different from zero (CI [− 16%, + 8%]), and the coefficient for at least moderate WTPA is negligible in magnitude.

In Specification 3, where the interaction between domains is considered and the PA dummies identify the effect of PA solely exerted in the respective domain, the coefficient for high LTPA indicates that women who are highly active during leisure time but low active in the travel and working domains have 22% (CI [+ 2%, + 46%]) more visits than women who are low active in all domains. It is noteworthy that by the negative coefficient of the respective interaction term “LTPA high x TTPA mod” moderate TTPA mitigates the positive effect of LTPA. The coefficient estimate for WTPA is significant at the 10% level, indicating 31% (CI [− 54%, + 5%]) fewer visits for women who are only active for working purposes compared to low active women. However, as only 27 women are in this group, these estimates should be interpreted with caution.

The Wald test rejects at the 10% level (*p* value = 0.075) the null hypothesis that the coefficient estimate for high LTPA in Specification 3 is not different from the estimate for high total PA in Specification 1. The estimates for moderate LTPA or moderate TTPA in Specification 3 in contrast are found not to be significantly different from the coefficient of moderate total PA in Specification 1 (*p* values 0.27 and 0.96, respectively).

### Men

For the male sample, none of the coefficients for either PA dummy is significant in any of the three specifications (Table 3). In Specification 1, as in the case of women, both coefficients for total PA are negligible. In contrast to women, both LTPA coefficients in Specification 2 are very low in magnitude, while the estimate for the WTPA coefficient is relatively high and indicates 15% fewer visits for men who are at least moderately active during working time. However, due also to large standard errors, the association is not significant (CI [− 36%, + 12%]). For men, all coefficients for physical activity in Specification 3 are negative for all domains and, except for high LTPA, relatively large. In particular being at least moderately active only during leisure time or during working time is associated with 17% (CI [− 45%, + 24%]) or 30% (CI [− 54%, + 8%]) fewer visits, respectively. However, again because of the large standard errors, the associations are not significant at the 10% level. The Wald test did neither detect a significant difference between the coefficients for moderate LTPA or moderate TTPA in Specification 3 and moderate total PA in Specification 1 (*p* values 0.31 and 0.44, respectively), nor between high LTPA in Specification 3 and high total PA in Specification 1 (*p* value 0.69). Also none of the sums of a PA dummy coefficient with the coefficient of the respective interaction term is significant.

## Discussion

With this study, we exploit the comprehensive assessment offered by the GPAQ questionnaire to analyze and compare the associations of PA with the number of contemporaneous physician visits, when physical activity is either considered with a measure which aggregates activity across domains or with a measure that differentiates between domains.

First our study highlights that gender differences in the association between PA and health care utilization are relevant and in this respect confirms findings of other studies where gender-specific effects are accounted for (Anderson et al. 2005; Gil et al. 2018; Yen 2012). In our study we found no significant association between either measure of PA and physician visits among the male participants of the Konstanz Life-Study. However, as suggested by the estimates in Specification 3, being active in one domain only is more likely to be negatively than positively associated with the number of visits for men. For women, in contrast, while no association can be detected with the aggregate measure either, domain-specific effects can be identified with the differentiated measure for LTPA: High LTPA is positively associated with the number visits, but this effect is mitigated for women who are also active during travel time. Further the assumption that the effect of physical activity in a single domain is not different from the effect of total PA, which is necessary for the aggregate measure to be appropriate, cannot be upheld at least for LTPA. Therefore, our results advocate the use of the differentiated measure rather than the aggregate measure for analyzing the association between PA and contemporaneous health care utilization.

The detailed assessment of physical activity in different domains however requires comparably long questionnaires (for the Konstanz Life-Study the GPAQ administered encompasses 16 questions) and might be too cumbersome for large household surveys. This is probably the reason why several studies are restricted to LTPA only (Arem et al. 2015; Carlson et al. 2015; Karl et al. 2018). Therefore, it might be interesting what our results would have looked like, if Specification 2 had been estimated with only including LTPA, but not TTPA and WTPA. It turns out that the coefficient estimates for moderate and high LTPA are not very different from the estimates with additionally TTPA and WTPA included (s. Table S3, supplementary material) suggesting that assessing LTPA only results in acceptable estimates for portraying the association of physical activity and physician visits.

In our study we found that high leisure time activity increases physician visits by more than 16% among women. Although this result is in contrast with the findings by Sari (2009), who analyzed Canadian data, it is consistent with the findings by Kang and Xiang (2017) for US-MEPS data, who report an increased odds ratio of having an office based visit for active individuals. Our findings are also consistent with two studies using data from the representative German Socio-Economic Panel, with one study reporting a significant (Eibich and Ziebarth 2014) and the other study reporting an insignificant positive association (Winkelmann 2004) of regular sport with the number of physician visits. However, none of these studies account for gender differences in the association of physical activity with physician visits. To clarify the external validity of our results, we defined a new dummy variable which indicates whether at least 60 min of vigorous activity per week is accumulated and which resembles the physical activity variable in the SOEP sample (Sport once a week). We regressed this variable on physician visits in a NegBin Model using the complete sample (men and women). The results indicate an insignificant increase by 5.6% of visits for “active” individuals (s. Table S3, supplementary material), which is very close to the insignificant increase of 4.6% reported by Winkelmann (2004).

We can only speculate on the reasons for this positive relationship between high LTPA and physician visits among the female participants of the Konstanz-Life study. Three explanations are commonly discussed. First, vigorous activity and sport are associated with increased risk for damage of body tissue and cardiovascular events (Verhagen et al. 2012). However, if this was a major reason here, the positive association should be evident also among men. Second, physically active people are reported to use preventive health care services and checkups more often than inactive people (Kang and Xiang 2017), maybe because they are more health conscious. However, also this explanation presumes that only women are affected by this association, but evidence suggests also a positive association between physical activity and prostate cancer screening (Kang and Xiang 2017). The third explanation can be seen in an unhealthy quest for lowering body fat among young women and the symptoms of so called female triad—a combination of eating disorders, amenorrhea and osteoporosis, which is observed among high-level female athletes—can also be found in a general active female population (Verhagen et al. 2012; Milano et al. 2020). This hypothesis is supported inasmuch as the positive association between high LTPA and visits is strongest among the younger female participants of the Konstanz-Life Study with low body mass index (s. Table S3, supplementary material). In any case, our results provides additional evidence that LTPA is not necessarily associated with lower health care utilization.

### Limitations

We are aware of several limitations of our study. First, due to data restrictions we are limited to analyzing the domain-specific effects of physical activity to physician visits only. But other kinds of health care services are affected by physical activity as well. In particular, those studies where a positive association with physician visits was found, at the same time report a negative association with inpatient visits (Kang and Xiang 2017; Eibich and Ziebarth 2014). Secondly, the mere number of physician visits does not allow inferring unambiguously the actual health care cost associated, as for example medications prescribed by the doctor may differ. However, among those female participants of the Konstanz Life Study who had a physician visit, no difference is evident in the number of medications, taken on a regular basis for thirteen medical conditions (hypertension, diabetes, sedative, etc.), when we compare women with high or moderate LTPA with those with low LTPA (not shown). Third, because of the cross-sectional data, the estimated associations between physical activity and physician visits may not reflect true causality because of unobserved factors like health consciousness. Longitudinal data would provide the most reliable way to control for such unobserved factors. But as longitudinal data are difficult and costly to acquire, much would be gained if further information on the diagnosis or the kind of treatment was regularly collected along cross-sectional studies, so that more could be learned about the causal effects of physical activity on contemporaneous health care utilization.

### Conclusions

Among the female participants of the Konstanz Life-Study, high physical activity during leisure time is associated with an increased number of contemporaneous physician visits. As this effect is different from the effect of high total physical activity, the results reject the aggregate measure to be appropriate for analyzing the relationship between physical activity and contemporaneous health care utilization. The results also suggest that possible positive effects on health care utilization should be taken into account in attempts to foster leisure time physical activity.

## Electronic supplementary material

Below is the link to the electronic supplementary material.

Supplementary material 1 (DOCX 29 kb)
