# Supplementary material for: Domain-specific effects of physical activity on the demand for physician visits
Source: Int J Public Health. 2020 May 6;65(5):583–91. doi: 10.1007/s00038-020-01376-5 (PMC7360656; doi:10.1007/s00038-020-01376-5)
Supplement: Supplementary file 1 — Supplementary material 1 (DOCX 29 kb) [file 38_2020_1376_MOESM1_ESM.docx]

**Supplemental tables to “Domain specific effects of physical activity on the demand for physician visits” / International Journal of Public Health**

Table S1 Estimation results negative binomial model, women. Dependent variable: number of physician visits. Data from the Konstanz Life-Study, waves 2016 and 2017, Germany.

| Independent variables | Specification 1 | Specification 2 | Specification 3 |
| --- | --- | --- | --- |
| Total PA (ref.: Low) |  |  |  |
| Moderate | -0.03 (0.10) | - | - |
| High | 0.04 (0.10) | - | - |
| LTPA (ref.: Low) |  |  |  |
| Moderate | - | 0.11 (0.09) | 0.11 (0.14) |
| High | - | 0.15* (0.07) | 0.20* (0.09) |
| TTPA (ref.: Low) |  |  |  |
| Moderate | - | -0.05 (0.06) | -0.02 (0.09) |
| WTPA (ref.: Low) |  |  |  |
| At least moderate | - | 0.01 (0.16) | -0.37+ (0.21) |
| Interactions |  |  |  |
| LTPA mod x TTPA mod | - | - | -0.02 (0.19) |
| LTPA high x TTPA mod | - | - | -0.15 (0.13) |
| LTPA mod x WTPA act | - | - | 0.09 (0.29) |
| LTPA high x WTPA act | - | - | 0.33 (0.33) |
| TTPA mod x WTPA act | - | - | 0.36 (0.29) |
| High school degree | -0.05 (0.07) | -0.05 (0.08) | -0.06 (0.08) |
| Age | 0.00 (0.00) | 0.00 (0.00) | 0.00 (0.00) |
| Occupation (ref. employed/self-employed) |  |  |  |
| Student/vocational training | -0.06 (0.13) | -0.04 (0.13) | -0.03 (0.12) |
| Unemployed | 0.37+ (0.21) | 0.36+ (0.20) | 0.35+ (0.20) |
| Retired | -0.19 (0.12) | -0.19 (0.13) | -0.22+ (0.13) |
| Homemaker | -0.33. (0.20) | -0.31 (0.20) | -0.29 (0.20) |
| Income (ref.: <1000 EUR / month) |  |  |  |
| 1000 - 2000 EUR / month | 0.01 (0.10) | 0.01 (0.10) | 0.01 (0.10) |
| 2000 - 3000 EUR / month | -0.14 (0.11) | -0.13 (0.11) | -0.13 (0.11) |
| 3000 - 5000 EUR / month | 0.06 (0.11) | 0.05 (0.11) | 0.06 (0.11) |
| > 5000 EUR / month | 0.11 (0.12) | 0.09 (0.12) | 0.10 (0.12) |
| Children < 14 years in household | -0.07 (0.10) | -0.06 (0.10) | -0.05 (0.10) |
| Health status (ref.: poor/very poor) |  |  |  |
| Fair | -0.28* (0.13) | -0.28* (0.13) | -0.28* (0.13) |
| Good/very good | -0.65*** (0.12) | -0.67*** (0.13) | -0.65*** (0.13) |
| Chron. disease | 0.50*** (0.07) | 0.51*** (0.07) | 0.51*** (0.07) |
| Waist-to-hip ratio | 0.85 (2.73) | 0.82 (2.64) | 0.43 (2.78) |
| Waist-to-hip ratio2 | -0.65 (1.50) | -0.62 (1.45) | -0.40 (1.54) |
| Smoker | 0.08 (0.09) | 0.08 (0.09) | 0.07 (0.09) |
| Alcohol consumption (ref.: <100 g alc/week) |  |  |  |
| 100 - 200 g alc/week | 0.05 (0.10) | 0.04 (0.10) | 0.04 (0.10) |
| 200 - 350 g alc/week | 0.22+ (0.12) | 0.22+ (0.13) | 0.21+ (0.12) |
| > 350 g alc/week | -0.15 (0.26) | -0.13 (0.26) | -0.14 (0.26) |
| Wave 2017 | -0.06 (0.06) | -0.06 (0.06) | -0.06 (0.06) |
| n | 1330 | 1330 | 1330 |
| Akaike Information Criterion | 4473.4 | 4473.9 | 4474.7 |

+ p < 0.1, * p < 0.05, ** p < 0.01, *** p < 0.001, cluster-robust standard errors in parentheses

LTPA: leisure time physical activity, TTPA: travel time physical activity, WTPA: working time physical activity

Table S2 Estimation results negative binomial model, men. Dependent variable: number of physician visits. Data from the Konstanz Life-Study, waves 2016 and 2017, Germany.

| Independent variables | Specification 1 | Specification 2 | Specification 3 |
| --- | --- | --- | --- |
| Total PA (ref.: Low) |  |  |  |
| Moderate | 0.02 (0.14) | - | - |
| High | 0.04 (0.14) | - | - |
| LTPA (ref.: Low) |  |  |  |
| Moderate | - | -0.04 (0.15) | -0.19 (0.21) |
| High | - | 0.03 (0.11) | -0.02 (0.16) |
| TTPA (ref.: Low) |  |  |  |
| Moderate | - | 0.05 (0.10) | -0.10 (0.15) |
| WTPA (ref.: Low) |  |  |  |
| At least moderate | - | -0.16 (0.14) | -0.35 (0.22) |
| Interactions |  |  |  |
| LTPA mod x TTPA mod | - | - | 0.23 (0.28) |
| LTPA high x TTPA mod | - | - | 0.14 (0.21) |
| LTPA mod x WTPA act | - | - | 0.48 (0.43) |
| LTPA high x WTPA act | - | - | -0.23 (0.31) |
| TTPA mod x WTPA act | - | - | 0.39 (0.27) |
| High school degree | 0.14 (0.10) | 0.12 (0.10) | 0.12 (0.10) |
| Age | 0.00 (0.01) | 0.00 (0.01) | 0.00 (0.01) |
| Occupation (ref. employed/self-employed) |  |  |  |
| Student/vocational training | 0.42* (0.20) | 0.41* (0.20) | 0.44* (0.20) |
| Unemployed | 0.31 (0.30) | 0.29 (0.30) | 0.32 (0.29) |
| Retired | 0.22 (0.18) | 0.24 (0.18) | 0.21 (0.18) |
| Homemaker | -0.06 (0.44) | -0.10 (0.44) | -0.06 (0.44) |
| Income (ref.: <1000 EUR / month) |  |  |  |
| 1000 - 2000 EUR / month | 0.06 (0.18) | 0.07 (0.18) | 0.08 (0.18) |
| 2000 - 3000 EUR / month | 0.21 (0.22) | 0.22 (0.23) | 0.23 (0.22) |
| 3000 - 5000 EUR / month | 0.13 (0.21) | 0.16 (0.22) | 0.17 (0.21) |
| > 5000 EUR / month | -0.11 (0.23) | -0.11 (0.23) | -0.09 (0.23) |
| Children < 14 years in household | -0.02 (0.14) | -0.03 (0.15) | -0.02 (0.14) |
| Health status (ref.: poor/very poor) |  |  |  |
| Fair | -0.53** (0.20) | -0.52** (0.20) | -0.52** (0.20) |
| Good/very good | -0.88*** (0.20) | -0.86*** (0.20) | -0.86*** (0.20) |
| Chron. disease | 0.76*** (0.11) | 0.76*** (0.11) | 0.76*** (0.11) |
| Waist-to-hip ratio | 1.84 (7.26) | 2.06 (7.35) | 1.65 (7.24) |
| Waist-to-hip ratio2 | -1.37 (3.79) | -1.47 (3.83) | -1.30 (3.77) |
| Smoker | -0.10 (0.12) | -0.11 (0.12) | -0.12 (0.12) |
| Alcohol consumption (ref.: <100 g alc/week) |  |  |  |
| 100 - 200 g alc/week | 0.25* (0.12) | 0.23+ (0.13) | 0.25* (0.12) |
| 200 - 350 g alc/week | 0.19 (0.21) | 0.18 (0.21) | 0.18 (0.20) |
| > 350 g alc/week | -0.07 (0.21) | -0.09 (0.21) | -0.03 (0.22) |
| Wave 2017 | -0.03 (0.10) | -0.02 (0.10) | -0.02 (0.10) |
| n | 766 | 766 | 766 |
| Akaike Information Criterion | 2227.2 | 2230.3 | 2235.5 |

+ p < 0.1, * p < 0.05, ** p < 0.01, *** p < 0.001, cluster-robust standard errors in parentheses

LTPA: leisure time physical activity, TTPA: travel time physical activity, WTPA: working time physical activity

Table S3 Estimation results negative binomial model with a) physical activity considered only for leisure time and b) physical activity defined as at least 60 minutes of vigorous activity per week during leisure time (similar to the definition in Winkelmann (2004)^a^ “active sports at least once a week”). Specification c) includes the interaction of leisure time physical activity with age/body mass index. The coefficient estimates for the interaction terms indicate that the positive association of high leisure time activity and the number of physician visits is strongest among younger women in the lowest quartile of body mass index. Dependent variable: number of physician visits. Data from the Konstanz Life-Study, waves 2016 and 2017, Germany.

| Independent variables | a) Only LTPA  women | b) Sport  Total sample | c) Interaction LTPA with age/BMI  women |
| --- | --- | --- | --- |
| LTPA (ref.: Low) |  |  |  |
| Moderate | 0.10 (0.09) | - | 0.21 (0.25) |
| High | 0.15* (0.07) | - | 0.25 (0.18) |
| Sport |  | 0.06 (0.06) |  |
| TTPA (ref.: Low) |  |  |  |
| Moderate | - | - | -0.06 (0.06) |
| WTPA (ref.: Low) |  |  |  |
| At least moderate | - | - | 0.01 (0.16) |
| BMI (ref.: BMI mid) |  |  |  |
| BMI low |  |  |  |
| BMI high |  |  |  |
| Age | 0.00 (0.00) | 0.00 (0.00) | 0.00 (0.00) |
| Interactions |  |  |  |
| LTPA mod x Age | - | - | 0.00 (0.01) |
| LTPA high x Age | - | - | -0.01 (0.00) |
| LTPA mod x BMI low | - | - | 0.10 (0.19) |
| LTPA high x BMI low | - | - | 0.34+ (0.20) |
| LTPA mod x BMI high | - | - | 0.01 (0.22) |
| LTPA high x BMI high | - | - | 0.15 (0.15) |
| High school degree | -0.05 (0.07) | 0.03 (0.06) | -0.06 (0.08) |
| Male | - | -0.33*** (0.07) | - |
| Occupation (ref. employed/self-employed) | |  |  |
| Student/vocational training | -0.05 (0.13) | 0.11 (0.11) | -0.04 (0.13) |
| Unemployed | 0.36. (0.20) | 0.29. (0.16) | 0.38+ (0.20) |
| Retired | -0.20 (0.13) | 0.03 (0.10) | -0.18 (0.13) |
| Homemaker | -0.30 (0.20) | -0.27 (0.18) | -0.32+ (0.20) |
| Income (ref.: <1000 EUR / month) |  |  |  |
| 1000 - 2000 EUR / month | 0.01 (0.10) | 0.04 (0.09) | 0.01 (0.10) |
| 2000 - 3000 EUR / month | -0.13 (0.11) | 0.03 (0.11) | -0.13 (0.11) |
| 3000 - 5000 EUR / month | 0.06 (0.11) | 0.09 (0.10) | 0.06 (0.11) |
| > 5000 EUR / month | 0.10 (0.13) | 0.03 (0.11) | 0.09 (0.13) |
| Children < 14 years in household | -0.06 (0.10) | -0.08 (0.08) | -0.06 (0.10) |
| Health status (ref.: poor/very poor) |  |  |  |
| Fair | -0.0364 | -0.36** (0.12) | -0.28* (0.13) |
| Good/very good | -0.67*** (0.12) | -0.73*** (0.11) | -0.67*** (0.13) |
| Chron. disease | 0.51*** (0.07) | 0.61*** (0.06) | 0.50*** (0.07) |
| Waist-to-hip ratio | 0.88 (2.70) | 0.63 (2.72) | 0.74 (2.65) |
| Waist-to-hip ratio2 | -0.66 (1.48) | -0.67 (1.48) | -0.60 (1.45) |
| Smoker | 0.08 (0.09) | 0.01 (0.08) | 0.08 (0.09) |
| Alcohol consumption (ref.: <100 g alc/week) | |  |  |
| 100 - 200 g alc/week | 0.04 (0.10) | 0.12 (0.08) | 0.05 (0.10) |
| 200 - 350 g alc/week | 0.22+ (0.13) | 0.20+ (0.12) | 0.22+ (0.12) |
| > 350 g alc/week | -0.14 (0.26) | -0.08 (0.16) | -0.10 (0.26) |
| Wave 2017 | -0.06 (0.07) | -0.04 (0.06) | -0.07 (0.06) |
| n | 1330 | 2096 | 1330 |
| Akaike Information Criterion | 4468.8 | 6700.2 | 4476.7 |

+ p < 0.1, * p < 0.05, ** p < 0.01, *** p < 0.001, cluster-robust standard errors in parentheses

^a^Winkelmann, R., 2004. Health Care Reform and the Number of Doctor Visits-An Econometric Analysis. Journal of Applied Econometrics 19, 455-472

LTPA: leisure time physical activity, TTPA: travel time physical activity, WTPA: working time physical activity, BMI: body mass index
